# Direct Detection of Diverse Metabolic Changes in Virally Transformed and Tax-Expressing Cells by Mass Spectrometry

**DOI:** 10.1371/journal.pone.0012590

**Published:** 2010-09-07

**Authors:** Prabhakar Sripadi, Bindesh Shrestha, Rebecca L. Easley, Lawrence Carpio, Kylene Kehn-Hall, Sebastien Chevalier, Renaud Mahieux, Fatah Kashanchi, Akos Vertes

**Affiliations:** 1 Department of Chemistry, W. M. Keck Institute of Proteomics Technology and Applications, The George Washington University, Washington, D. C., United States of America; 2 Department of Biochemistry and Molecular Biology, The George Washington University School of Medicine, Washington, D. C., United States of America; 3 Department of Molecular and Microbiology, National Center for Biodefense and Infectious Diseases, George Mason University, Manassas, Virginia, United States of America; 4 Laboratory of Cellular Oncology, National Institutes of Health/National Cancer Institute, Bethesda, Maryland, United States of America; 5 Equipe Oncogenèse Rétrovirale, Ecole Normale Supérieure de Lyon, U758 INSERM, Lyon, France; National Institutes of Health, United States of America

## Abstract

**Background:**

Viral transformation of a cell starts at the genetic level, followed by changes in the proteome and the metabolome of the host. There is limited information on the broad metabolic changes in HTLV transformed cells.

**Methods and Principal Findings:**

Here, we report the detection of key changes in metabolites and lipids directly from human T-lymphotropic virus type 1 and type 3 (HTLV1 and HTLV3) transformed, as well as Tax1 and Tax3 expressing cell lines by laser ablation electrospray ionization (LAESI) mass spectrometry (MS). Comparing LAESI-MS spectra of non-HTLV1 transformed and HTLV1 transformed cells revealed that glycerophosphocholine (PC) lipid components were dominant in the non-HTLV1 transformed cells, and PC(O-32∶1) and PC(O-34∶1) plasmalogens were displaced by PC(30∶0) and PC(32∶0) species in the HTLV1 transformed cells. In HTLV1 transformed cells, choline, phosphocholine, spermine and glutathione, among others, were downregulated, whereas creatine, dopamine, arginine and AMP were present at higher levels. When comparing metabolite levels between HTLV3 and Tax3 transfected 293T cells, there were a number of common changes observed, including decreased choline, phosphocholine, spermine, homovanillic acid, and glycerophosphocholine and increased spermidine and N-acetyl aspartic acid. These results indicate that the lipid metabolism pathway as well as the creatine and polyamine biosynthesis pathways are commonly deregulated after expression of HTLV3 and Tax3, indicating that the noted changes are likely due to Tax3 expression. N-acetyl aspartic acid is a novel metabolite that is upregulated in all cell types and all conditions tested.

**Conclusions and Significance:**

We demonstrate the high throughput in situ metabolite profiling of HTLV transformed and Tax expressing cells, which facilitates the identification of virus-induced perturbations in the biochemical processes of the host cells. We found virus type-specific (HTLV1 vs. HTLV3), expression-specific (Tax1 vs. Tax3) and cell-type–specific (T lymphocytes vs. kidney epithelial cells) changes in the metabolite profiles. The new insight on the affected metabolic pathways can be used to better understand the molecular mechanisms of HTLV induced transformation, which in turn can result in new treatment strategies.

## Introduction

Human T-lymphotropic virus type 1 (HTLV1), a member of the delta-retroviridae subfamily, was the first human pathogenic retrovirus discovered and found to contribute to cancer development [1], [2]. Infection with HTLV1 has been shown to result in the development of adult T-cell leukemia (ATL), a CD4^+^ T lymphoproliferative malignancy. Estimates of worldwide HTLV1 infections are currently 15 to 25 million individuals. However, infected individuals develop ATL after a long latent period and at a 3-5% incidence rate. Evidence has also linked HTLV1 infection with HTLV1-associated myelopathy/tropical spastic paraparesis (HAM/TSP; [3]) and several inflammatory diseases including polymyositis [4], uveitis [5], and lymphocyte alveolitis [6]. The development of ATL from HTLV1 infection is thought to be a multi-hit occurrence with initial transformation due to the viral protein Tax1. Recent studies have indicated that the use of novel treatments, including monoclonal antibodies against the interleukin-2 receptor (IL-2R) and the combination therapy of interferon-alpha (IFN-α) and zidovudine (AZT), to be effective, but only in a small percentage of ATL patients. Therefore, new therapies are needed for the treatment of ATL or more specifically, HTLV1 infection.

Viral-induced transformation causes extensive changes at the gene, protein and metabolite levels. These changes are usually followed by gene-expression profiling and proteomic analysis [7], [8]. Exploring the metabolic consequences of viral transformation adds to the picture because the viruses rely on the metabolic network of their cellular hosts for survival and replication [9], [10], [11]. Genomic, proteomic and metabolomic technologies have not only provided the foundation for the enhanced understanding of cell biology, but they are also emerging as tools for identifying disease biomarkers and for drug development. When compared to the transcriptome and the proteome, monitoring of the metabolome is useful because the metabolic composition of a cell/tissue provides its actual biochemical condition. Although metabolic technologies have been applied to find biomarkers for a few cancerous and virally transformed cell types [9], [12], [13], [14], [15], these techniques need to be expanded for challenging viral transformations, such as human immunodeficiency virus (HIV) and HTLV1.

The insight gained by such studies depends on the target sample, the treatment procedures and the detection techniques used. Conventionally, biofluids such as blood and urine have been used to follow metabolic changes after infection, but in many cases they complicate the analysis due to the pooling of changes in different cell types and the variations between individuals [16], [17], [18]. Ultimately, direct analysis of cells/tissues is a more straightforward way to understand the actual disease-associated metabolic changes occurring. In such cases, a direct detection technique offers key advantages.

Metabolites are small molecules of diverse physico-chemical properties with greatly different abundance levels that make their analysis challenging. Typically optical (e.g., Fourier transform infrared spectrometry), nuclear magnetic resonance (NMR) [19], [20], [21] and mass spectrometric techniques in combination with separation techniques, such as gas chromatography, high performance liquid chromatography (HPLC) and capillary electrophoresis, have been used for metabolomic studies [22], [23], [24], [25], [26]. Mass spectrometry (MS) is a technology with high efficiency [14] that, combined with chromatographic separations, can provide qualitative and quantitative analyses of complex samples with high selectivity and sensitivity, as well as a broad dynamic range. Conventional mass spectrometric methods, e.g., matrix-assisted laser desorption ionization (MALDI) and electrospray ionization (ESI), however, are time consuming because they involve extensive sample preparation steps [27]. The application of direct sampling methods, such as flow injection ESI, can avoid chromatographic separation [28], but not extensive sample preparation that can affect sample integrity and, in some cases, may lead to metabolite degradation. Therefore, many of these techniques restrict the choice of samples and preclude their in situ analysis. Some of these problems can be mitigated by the use of atmospheric pressure ion sources.

Recent advances in atmospheric pressure ion sources, such as direct analysis in real time (DART) [29], [30], desorption electrospray ionization (DESI) [31], atmospheric pressure infrared MALDI (AP IR-MALDI) [32], [33], [34], [35] and laser ablation electrospray ionization (LAESI) [36], [37], [38] enabled direct analysis of tissues and cell samples without sample preparation. Analysis of cells, cell cultures and cell extracts using DART, DESI and MALDI techniques are known [39], [40], [41] but these methods have their own limitations, such as coverage of analytes, sampling of the surface only, and quantitation restrictions. The LAESI technique, developed for in situ tissue and cell analysis, samples the entire volume of the cells for metabolites and lipid components with tissue imaging and quantitation capabilities [42], [43].

Through the analysis and molecular imaging of various tissue samples, we have demonstrated that with LAESI it is possible to simultaneously detect different classes of compounds, for example, acidic and basic compounds, lipids and fatty acids [44], [45]. Relative quantitation of metabolites can be performed by directly comparing the LAESI signal from different samples [36], whereas absolute quantitation can be carried out using labeled internal standards [38]. A limitation of the LAESI technique is that it relies on the water content of the sample for analysis. Thus samples without intrinsic water can only be analyzed after wetting them. Similar to ESI, the LAESI technique is prone to matrix effects. Consequently, low-abundant metabolites can be missed or masked in the presence of matrix peaks or other highly abundant components.

In this contribution we aim to identify metabolic changes in HTLV1 transformed T lymphocytes by the direct application of the LAESI method that is rapid and eliminates the need for sample preparation. We focus on changes related to HTLV induced transformation or Tax expression that alter the metabolic profiles in different cell lines. Understanding the role of Tax in destabilizing key regulators, such as proteins in metabolism and cell cycle control, may help to identify molecular markers that contribute to ATL development and define new therapeutic strategies. We demonstrate that in situ metabolite profiling of HTLV1 transformed T lymphocytes facilitates the identification of virus-induced perturbations in the biochemical processes of the host cell. For comparison purposes, we performed similar experiments on cells transfected with either the HTLV3 molecular clone or Tax3 and compared these metabolic profiles with the results for HTLV1 transformed and Tax1 expressing cells.

## Materials and Methods

### Materials

The non-HTLV transformed T lymphocyte cells (CEM and H9) and kidney epithelial cells (293T) ), the HTLV1 transformed cells (C81 and HUT102), the H9 cells stably transfected with Tax1 of HTLV1 (H9-Tax1) and the 293T cells transfected with HTLV3 (293T-HTLV3) and expressing Tax3 (293T-Tax3) were maintained in RPMI 1640 medium containing fetal bovine serum, L-glutamine (2 mM), penicillin (100 units/ml) and streptomycin (100 µg/ml). All the medium solutions and buffers were procured from Quality Biological Inc. (Gaithersburg, MD). All solvents used for MS were HPLC grade from Acros Organics (Geel, Belgium). The glacial acetic acid was purchased from Fluka (Munich, Germany).

### Laser Ablation Electrospray Ionization

Laser ablation was performed by a mid-IR laser system. An optical parametric oscillator (OPO) (Opolette 100, Opotek, Carlsbad, CA) converted the output of a 100-Hz repetition rate Nd∶YAG laser to mid-IR pulses of 5-ns duration at 2940-nm wavelength. Beam steering and focusing were accomplished by gold coated mirrors (PF10-03-M01, Thorlabs, Newton, NJ) and a 150 mm focal length CaF_2_ lens (Infrared Optical Products, Farmingdale, NY), respectively. At ∼5–6 mm downstream from the tip of the spray capillary, the laser beam with average output energy of 0.3 mJ/pulse was used to ablate the tissue sample at right angle. Optical microscopy of the burn pattern produced on a photographic paper indicated that the laser spot size had ∼300 µm diameter.

The electrospray system was similar to the one described in recent reports from our laboratory [36], [37], [38]. Briefly, a home-built electrospray system with a low-noise syringe pump (Physio 22, Harvard Apparatus, Holliston, MA) was used to feed the 50% methanol solution containing 0.1% (v/v) acetic acid through a stainless steel emitter with 320 µm o.d. and a tapered tip of 50 µm i.d. (MT320-50-5-5, New Objective Inc., Woburn, MA). Stable high voltage (2800 V) was generated by a regulated power supply (PS350, Stanford Research Systems, Inc., Sunnyvale, CA) and was directly applied to the emitter. The orifice of the sampling cone was on-axis with the electrospray emitter at a distance of 12 mm from its tip.

The cells of interest were grown to a similar population size (to produce ∼10^6^ cells/pellet) before subjecting them to LAESI experiments. The cells were quickly washed twice with phosphate buffered saline (PBS) and pelleted by spindown (2000 rpm). The supernatant PBS was completely removed without disturbing the pellet and a ∼10 µl fraction of the pellet was loaded onto a microscope slide for direct LAESI analysis. Under our experimental conditions, the actual number of cells ablated during LAESI analysis is much lower (∼2800 cells/laser shot). The microscope slide with the cell pellet was held at ambient temperature and was positioned 15 mm below the spray axis. The microscope slide was mounted on a computer-controlled stepper motor-driven three-axis precision flexure stage (Nanomax TS, Thorlabs, Newton, NY) for rastering and geometry optimization.

The LAESI ion source was mounted on a Q-TOF Premier mass spectrometer (Waters, Milford, MA). Full scan mass spectra were recorded over the mass range of m/z 50–2,000 using a time-of-flight (TOF) analyzer at a resolution of 8,000 (FWHM). Individual measurements on the T-cells took a few seconds. For structure identification of the metabolites, collision induced dissociation spectra were recorded by selecting the precursor ion using a quadrupole analyzer (transmission window 2 Da) and the product ions were resolved by the TOF analyzer. Argon was used as the collision gas at a typical collision cell pressure of 4×10^−3^ mbar, and a collision energy set between 5 and 25 eV. Accurate masses were determined using the internal standard method. Glycine, methionine, N-acetyl phenylalanine, leucine enkephalin and glufibrinopeptide were dissolved at the appropriate concentrations (50–200 µM) in the electrospray solution and used as internal standards. Averages of the LAESI spectra collected under similar experimental conditions for a fixed time window were considered so that the approximate number of cells used for obtaining LAESI spectra were the same for all the studied cell types.

The human metabolome database (HMDB; www.hmdb.ca), the MassBank high resolution mass spectral database (www.massbank.jp), the NIST/EPA/NIH mass spectral library, and the MetaCyc database (http://metacyc.org) were used with a mass tolerance ranging from 0.1 to 0.01 Da for the metabolite searches and identifications.

### Enzyme assays

Arginase activity was measured using the QuantiChrom Arginase Assay Kit (BioAssay Systems, Hayward, CA) according to the manufacturer's instructions. CEM and C81 cell lysates (10 and 100 µg) were measured in triplicates. The concentration of cAMP was measured using the CatchPoint Cyclic-AMP Fluorescent Assay Kit (Molecular Devices, Sunnyvale, CA) according to the manufacturer's instructions. CEM and C81 cell lysates (10 and 100 µg) were measured in triplicates. Glutathione reductase from CEM and C81 cell lysates (10 and 200 µg) was measured utilizing the Glutathione Reductase Assay Kit (Sigma, St. Louis, MO) according to the manufacturer's instructions.

## Results

### CEM and C81 cells

**Figure 1:**
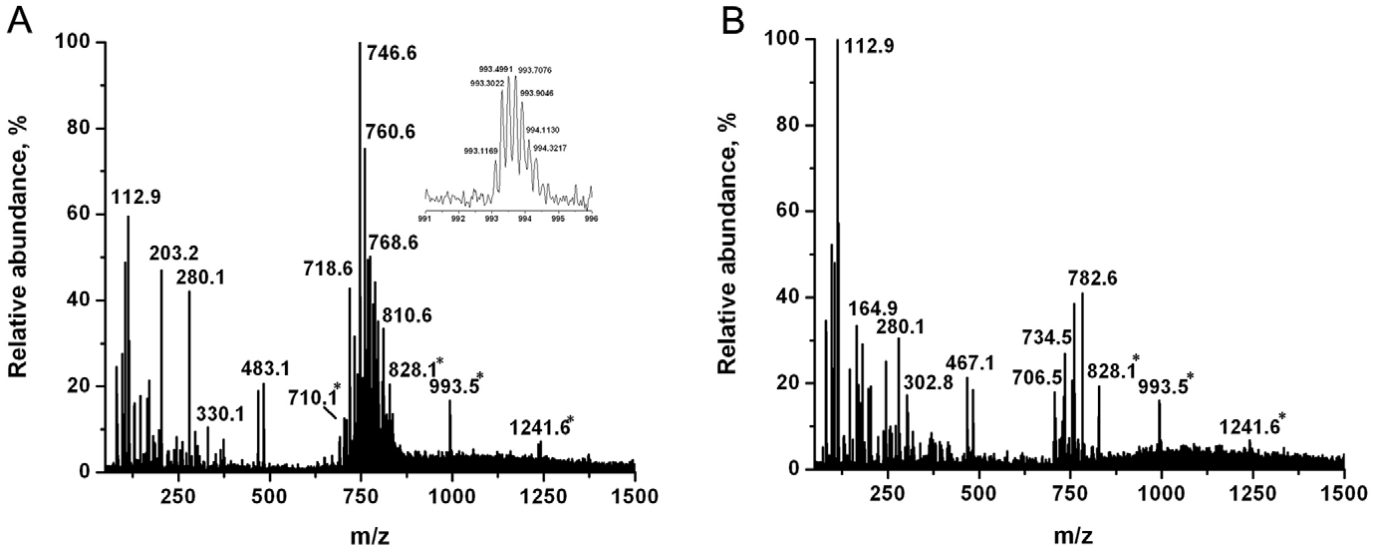
Positive ion LAESI mass spectra of T lymphocytes. Peaks marked in A) non-HTLV1 transformed CEM T lymphocytes and B) HTLV1 transformed C81 T lymphocytes with an asterisk (*) indicate multiply charged ions of a peptide. Inset in panel A) shows the isotope pattern of the peak at m/z 993.5 with five charges.

The initial experiments focused on populations of non-HTLV1 transformed (CEM) and HTLV1 transformed (C81) T lymphocytes. These non-adherent cells were grown in RPMI medium composed of inorganic salts, sugar, amino acids, vitamins and antibiotics. To minimize the interfering peaks from the medium in the LAESI spectra, the cells were quickly washed with PBS and the cell pellet was loaded onto a microscope slide. The cells were directly ablated by multiple laser shots and the average LAESI spectra (10–15 scans) were used for studying the metabolic changes. The resulting positive ion spectra exhibited various cell related metabolite ions in the range of m/z 20–1500, but also included a few interfering peaks from the PBS and the medium left in the cell pellet. Typical LAESI spectra obtained from CEM and C81 cells are shown in Figure 1. The metabolite peaks observed in the spectra were identified based on the accurate masses, isotope distribution patterns and structural information obtained from tandem MS. The background corrected spectra recorded from the cells consisted of protonated, sodiated and potassiated species. The observed peaks were due to small metabolites (<m/z 500), lipids (between m/z 690 and 850) and multiply charged peaks between m/z 700 and 1300. Deconvolution of all multiply charged peaks (m/z 710, 828, 993 and 1241) revealed that they corresponded to a single species with a nominal molecular weight of 4960.6, probably related to a peptide.

**Figure 2:**
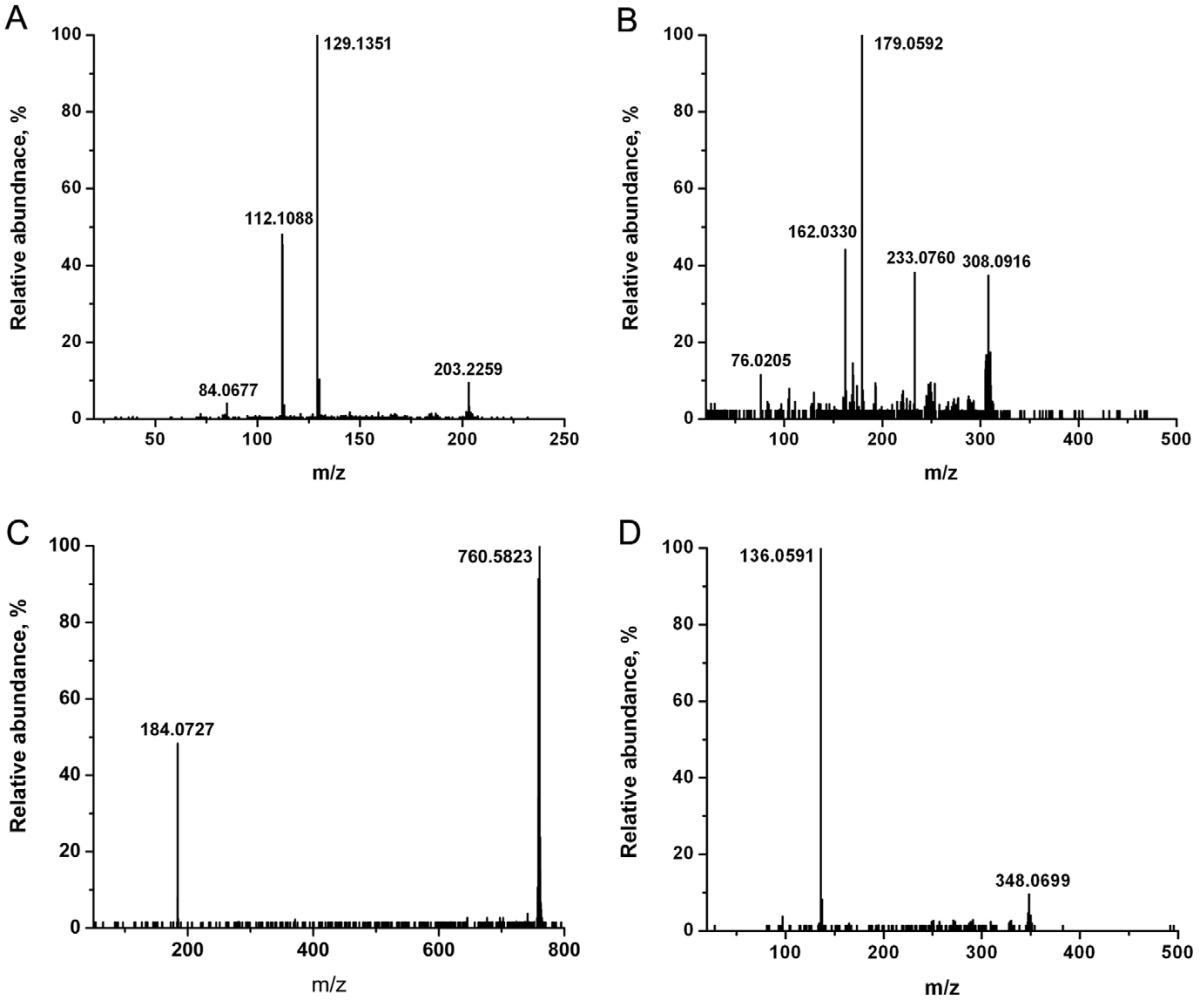
Tandem MS with collision induced dissociation for metabolite identification. A) protonated spermine (m/z 203.2), B) protonated glutathione (m/z 308.1) and C) protonated glycerophosphocholine lipid (PC(34:1), m/z 760.6) in CEM T lymphocytes, and of D) protonated adenosine monophosphate (AMP, m/z 348.1) in C81 T lymphocytes were directly analyzed by LAESI.

**Figure 3:**
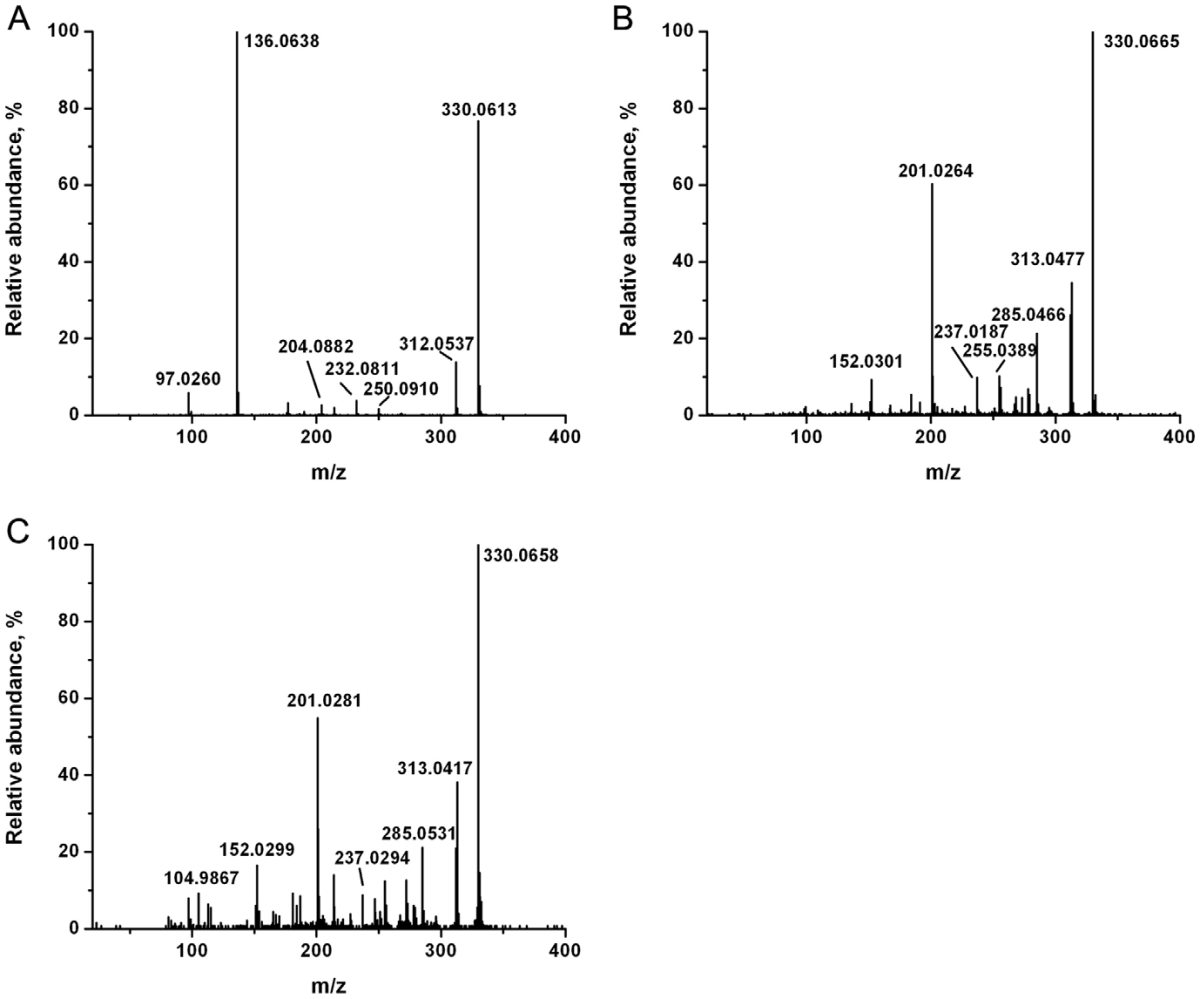
Tandem MS of m/z 330 obtained from standards and T lymphocytes. A) cyclic AMP standard (protonated), B) glutathione standard (sodiated) and C) CEM T lymphocytes were analyzed by LAESI and CID. Panels A) and B) show that the fragment ion m/z 136 is specific to AMP, whereas the fragment ions m/z 313, 285, 201 and 152 are specific to the sodiated glutathione. Thus the m/z 330 ion produced from CEM T lymphocytes in panel C) can be attributed to sodiated glutathione.

The spectra of CEM and C81 cells showed a similar set of ions, except for the lipid peaks, but consistent differences could be identified in their relative ion yields. The detected cell-specific metabolite ions and their peak assignments, based on accurate mass and tandem mass spectral data, are summarized in Table 1. All the key metabolites were confirmed by comparing their tandem mass spectra with the spectra of the corresponding standards or with spectra from tandem MS databases. The detected structure-specific fragment ions are listed in Table 1. Typical tandem mass spectra, used to identify spermine (m/z 203.2), glutathione (m/z 308.1), a phosphocholine lipid (PC(34∶1), m/z 760.6) and adenosine monophosphate (m/z 348.1), are shown in Figure 2. Theoretically for the m/z 330.0738 ion two structures can be assigned, namely protonated cyclic AMP (cAMP) or sodiated glutathione. Even if both the ions are contributing to m/z 330 (as tested with standards), it is impossible to distinguish these two ions with the available mass resolution (m/Δm ≈10,000 compared to the necessary 25,000). Comparing the tandem mass spectrum of the m/z 330 ion from the T lymphocytes with the tandem MS of the m/z 330 ions generated from the two standards revealed that the ion in the cell spectra was a sodiated glutathione (Figure 3). However, contribution of cAMP below the levels required for obtaining a tandem mass spectrum cannot be ruled out. In fact, cAMP was reported in T lymphocytes at the levels of 6 pmoles/10^7^ cells. The overwhelming interference from glutathione may be the cause of the difficulty in confirming the presence of cAMP by tandem MS under the experimental conditions used.

**Table 1:** List of metabolites detected in non-HTLV1 transformed (CEM, H9), and HTLV1 transformed (C81) and Tax1 expressing (H9-Tax1) T cells.

No.	Metabolite (chemical formula)	Ion	Measured mass*	Error (mDa)	Tandem MS fragment ions	Abundance Ratios* (CEM, C81)		Abundance Ratios (H9, H9-Tax1)	
						Up#	Down#	Up	Down
1	Thioacetamide (C_2_H_5_NS)	[M+H]+	76.0236	1.5		-	2.4 (0.6)	-	-
2	Putrescine (C_4_H_12_N_2_)	[M+H]+	89.1103	2.4		3 (2)	-	3.2	
	Pyrrolidine/Degradation product a, (C_4_H_9_N)	[M+H]+	72.0798	−1.5		2.1 (0.1)			
3	Choline (C_5_H_14_NO)	[M+H]+	104.1087	1.2	60,58	-	1.9 (0.5)	-	2.9
4	Proline (C_5_H_9_NO_2_)	[M+H]+	116.0709	−0.3	70	-	2.1 (1.1)	-	10.1
		[M+Na]+	138.0564	3.3					
		[M+K]+	154.0299	2.9					
5	Taurine (C_2_H_7_NO_3_S)	[M+H]+	126.0259	3.4		-	2.9 (0.6)	-	1.7
		[M+Na]+	147.9997	−4.7					
6	Creatine (C_4_H_9_N_3_O_2_)	[M+H]+	132.0816	4.3	90	4.6 (2.3)	-	2.3	-
7	Spermidine (C_7_H_19_N_3_)	[M+H[^+^	146.1664	0.7	72,112,129	1.7 (0.4)	-	1.0	-
8	p-Aminobenzoic acid (C_7_H_7_NO_2_)	[M+Na]+	160.039	1.6		2.7 (0.4)	-	-	1.7
9	Iminoaspartic acid (C_4_H_5_NO_4_)	[M+K]+	169.9866	1		-	1.6 (0.6)	-	6.0
10	Arginine (C_6_H_14_N_4_O_2_)	[M+H]+	175.1171	−2.4	70,116 130,158	3.4 (2.2)	-	1.2	-
11	Dopamine (C_8_H_11_NO_2_)	[M+Na]+	176.074	5.3		3.0 (0.3)	-	-	1.4
		[M+K]+	192.0414	−1.3					
12	Phosphocholine (C_5_H_14_NO_4_P)	[M+H]+	184.0767	2.8	86	-	4.8 (3.3)	-	4.1
13	Carbamoyl-phosphate (CH_4_NO_5_P)	[M+2Na-H]+	185.9544	1.4		-	2 (0.2)	-	3.5
14	Spermine (C_10_H_26_N_4_)	[M+H]+	203.2259	2.3	112,129	-	2.3 (0.9)	-	1.8
	Degradation product c (C_7_H_13_N)	[M+H]+	112.1068	−4.0	84		1.7 (0.2)		
	Degradation product b (C_7_H_16_N_2_)	[M+H]+	129.1422	3.0	112, 84		1.7 (0.4)		
15	Methoxytyramine (C_9_H_13_NO_2_)	[M+K]+	206.0537	−4.6		-	5.3 (1.7)	-	3.7
16	N-acetyl aspartic acid/N-formyl glutamic acid (C_6_H_9_NO_5_)	[M+K]+	214.0088	−3		2.1 (0.5)	-	1.7	-
17	Homovanillic acid (C_9_H_10_O_4_)	[M+K]+	221.0208	−0.8		3.5 (1.3)	-	1.3	-
18	Glycerophosphocholine (C_8_H_20_NO_6_P)	[M+H]+	258.1124	1.7	104	-	2.4 (1)	-	6.1
		[M+Na]+	280.0961	3.5					
		[M+K]+	296.0724	5.9					
19	Glutathione (C_10_H_17_N_3_O_6_S)	[M+H]+	308.0904	−1.2	162,179, 233	-	4.6 (1.5)	-	9.8
		[M+Na]+	330.0738	0.2					
		[M+2Na-H]+	352.0518	−3.7					
		[M+Na+K-H]+	368.0315	2					
		[M+3Na-2H]+	374.0388	1.3					
		[M+2Na+K-2H]+	390.0125	1.1					
20	8-Hydroxyguanosine (C_10_H_13_N_5_O_6_)	[M+K]+	338.053	2.7		-	1.9 (1)	-	-
21	Adenosine monophosphate (C_10_H_14_N_5_O_7_P)	[M+H]+	348.0712	0.3	136	7.6 (1.2)	-	3.1	-
		[M+Na]+	370.0505	−2.4	158				
		[M+2Na-H]+	392.0298	−5					

Up and down regulation is followed by abundance ratios.

# The values in parentheses are standard deviation from triplicate experiments.

*Unassigned ions were detected at m/z 158.1572 (2.5), 228.0363 (2.6), 260.0298 (3.6), 311.9216 (1.5), 333.9604 (2.2), and 346.0616 (1.2), where the values in parenthesis were the abundance ratio C81/CEM.

**Figure 4:**
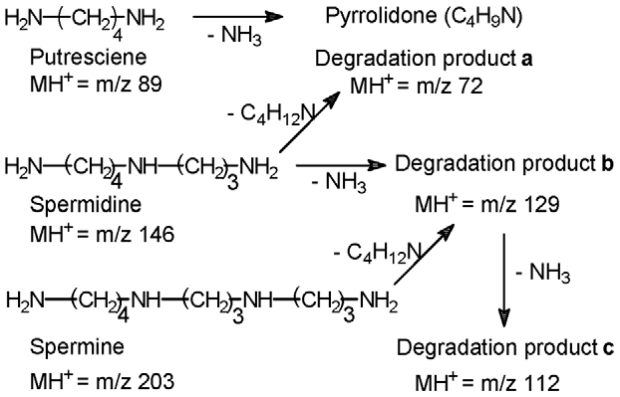
Degradation of putrescine, spermidine and spermine under LAESI experimental conditions.

Degradation/fragmentation products of putrescine, spermidine and spermine (Figure 4) were observed in the spectra and designated as degradation products **a**, **b** and **c** in Table 1. Formation of these products was confirmed based on the comparison of the LAESI data from standard polyamines with those detected in T lymphocytes at similar experimental conditions. The ion m/z 72 primarily results from putrescine, and m/z 129 and 112 can be formed from both spermidine and spermine, probably mostly from the latter due to its higher ion yields. The abundances of these degradation products between the non-HTLV1 transformed and HTLV1 transformed cells track those of their precursors.

**Figure 5:**
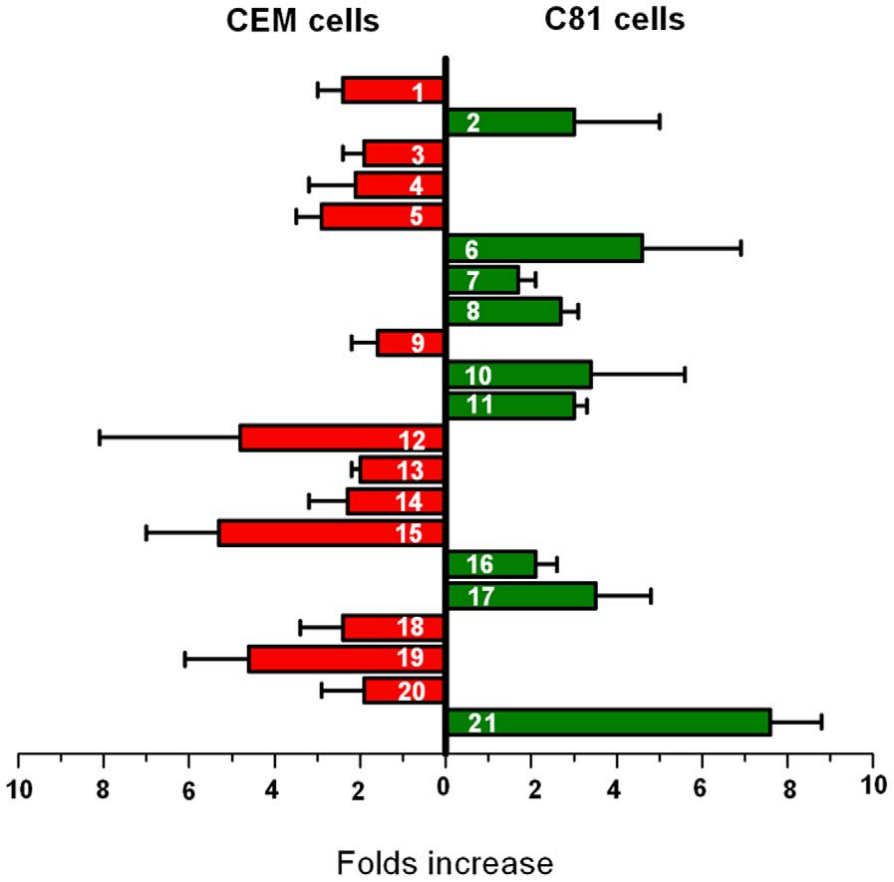
Metabolic differences between CEM and C81 cells detected by LAESI-MS.

In previous reports, we had demonstrated that LAESI spectra provided relative quantitation in a wide dynamic range [36], [38]. Hence, the relative abundances of the detected ions in the LAESI spectra of non-HTLV1 transformed and HTLV1 transformed cells were used to gauge the extent of metabolic changes between them. The background peaks from PBS/medium solution were used as internal standards to improve the accuracy of mass calibration. The relative abundance ratios for each ion detected in CEM and C81 are listed in Table 1. Some metabolites were detected as more than one ionic species (protonated, sodiated and potassiated). For example, glutathione was detected as six different ionic species. In such cases, the sum of the relative abundances of all the related species was used to calculate the abundance ratio. In case a particular peak was absent in a spectrum, the background (base line signal) was used to calculate the ratio. Upregulation was measured by the abundance ratio of ions from HTLV1 transformed over non-HTLV1 transformed cells, whereas downregulation was measured by the inverse ratio. A ratio close to 1.0 signified no change. The changes in the levels of the detected metabolites between CEM and C81 cells from triplicate experiments are shown in Figure 5.

Traces of glucose (relative abundance <3%), the major component of the medium (11 mM), appeared in the LAESI spectra of the T lymphocytes. The mass of protonated spermine (203.2236) was close to that of sodiated glucose species (203.059), but these two peaks were well separated. This, however, raised the issue of possible contribution of medium-related peaks to the spectra detected from T lymphocytes. The LAESI spectrum of the medium alone showed arginine (m/z 175), choline (m/z 104) and glutathione (m/z 308) that potentially contributed to the signal from the related metabolites in T lymphocytes. The glutathione and choline peaks were less than <2% with respect to the glucose peak (m/z 203, base peak), whereas the arginine peak was approximately 25–30%. These ratios were consistent with values from diluted medium (100 times). The glucose peak appeared in both CEM and C81 cells (<3%) with an abundance ratio for m/z 203 close to unit value. The arginine peak that was found to be negligible in CEM cells was much higher than the glucose peak in C81 cells. This confirmed that the arginine interferences from the medium were negligible and the arginine levels were indeed upregulated in C81 cells.

### Metabolic changes

Among the detected ions in the low mass region (<m/z 500), after correcting for the medium and electrospray related background peaks, there were about 43 ions exclusively related to T lymphocytes. Out of the 43 ions, 37 corresponded to 21 metabolites as seen in Table 1. The unassigned ions showing variations in their relative abundances between the non-HTLV1 transformed and the HTLV1 transformed cells are included in the footnote of Table 1. Many metabolites were downregulated in the HTLV1 transformed cells, e.g., spermine, choline, phosphocholine, glycerophosphocholine, and glutathione, whereas the levels of pyrrolidine, creatine, arginine, dopamine and adenosine monophosphate were upregulated.

### Lipid levels

**Figure 6:**
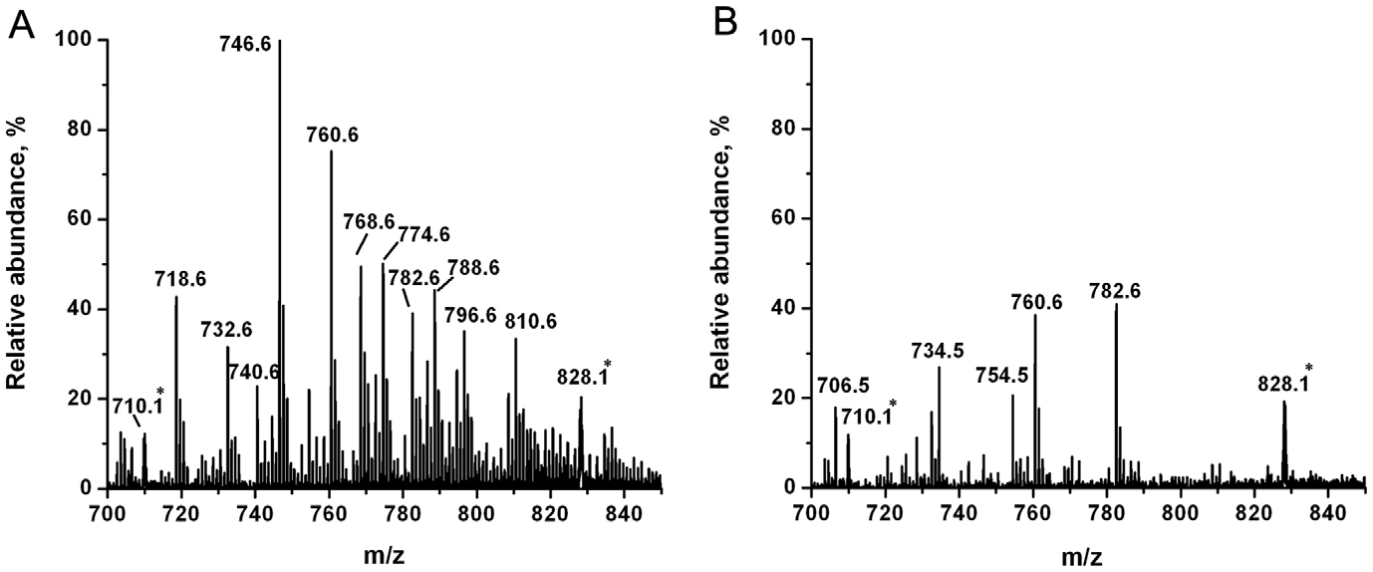
Expanded positive ion LAESI mass spectra showing glycerophosphocholine lipid peaks. Peaks marked with an asterisk (*) in A) CEM T lymphocytes B) HTLV1 transformed C81 T lymphocytes indicate multiply charged ions of a peptide.

We detected several glycerophosphocholine (PC) lipids in the LAESI spectra of the T lymphocytes. Dramatic changes were observed in the lipid abundances and types between non-HTLV1 transformed and HTLV1 transformed cells (expanded spectra are shown in Figure 6). Tandem mass spectra of all major lipid peaks yielded a single product ion at m/z 184 (a typical spectrum of the m/z 760.6 ion is shown in Figure 2C) that confirmed that they belong to PC lipids. Based on tandem mass spectrometric and accurate mass information, the detected lipid peaks were tentatively assigned as listed in Table 2. The table includes diacyl glycerophosphocholines (PC(C_n_∶db_n_), where C_n_  =  the total number of carbons and db_n_  =  the total number of double bonds in two fatty acid side chains, and alkylacyl/alkenylacyl glycerophosphocholines or plasmalogens, (PC(O-C_n_∶db_n_)). The measured relative abundance ratio values are also incorporated in Table 2. Most lipids detected in non-HTLV1 transformed were downregulated in HTLV1 transformed cells. Only a few lipids were retained in the HTLV1 transformed cells, and the levels of PC(30∶0), PC(O-31∶2), PC(32∶3), PC(32∶0), and PC(O-33∶3) were found to be higher compared to non-HTLV1 transformed cells.

**Table 2:** Lipid peaks detected in the LAESI spectra of non-HTLV1 transformed (CEM), and HTLV1 transformed (C81) T cells.

Sl. No.	Lipid*	Chemical formula	Ion	Monoisotopic mass	Observed mass	Error (mDa)	MS/MS	Abundance ratio	
								C81/CEM	CEM/C81
1	PC(29:1)	C_37_H_72_NO_8_P	[M+H]+	690.5074	690.5145	7.1			7.4
2	PC(O-30:0)	C_38_H_78_NO_7_P	[M+H]+	692.5594	692.5631	3.7			3.8
3	PC(O-31:2)	C_39_H_76_NO_7_P	[M+H]+	702.5438	702.5402	−3.6			7.7
4	PA O-37:1)	C_40_H_79_O_7_P	[M+H]+	703.5642	703.5730	8.8			2.4
5	PC(O-31:1)	C_39_H_78_NO_7_P	[M+H]+	704.5594	704.5579	−1.5			2.6
6	PC(30:0)	C_38_H_76_NO_8_P	[M+H]+	706.5387	706.5424	3.7	184	1.7	
7	PC(O-30:0)	C_38_H_78_NO_7_P	[M+Na]+	714.5414	714.5346	−6.8			2.7
8	PC(O-32:1)	C_40_H_80_NO_7_P	[M+H]+	718.5751	718.5652	−9.9	184		18.9
9	PC(O-32:0)	C_40_H_82_NO_7_P	[M+H]+	720.5907	720.5859	−4.8			2.9
10	PC(O-31:2)	C_39_H_76_NO_7_P	[M+Na]+	724.5257	724.5173	−8.4		1.1	
11	PA(O-37:1)	C_40_H_79_O_7_P	[M+Na]+	725.5461	725.5467	0.6			1.6
12	PC(32:3)	C_40_H_74_NO_8_P	[M+H]+	728.523	728.5212	−1.8		1.5	
13	PC(32:2)	C_40_H_76_NO_8_P	[M+H]+	730.5387	730.5436	4.9			2.7
14	PC(32:1)	C_40_H_78_NO_8_P	[M+H]+	732.5543	732.5537	−0.6			1.8
15	PC(32:0)	C_40_H_80_NO_8_P	[M+H]+	734.57	734.5748	4.8	184	2	
16	PC(O-32:1)	C_40_H_80_NO_7_P	[M+Na]+	740.5570	740.5496	−7.4			8.3
	PC(O-34:4)	C_42_H_78_NO_7_P	[M+H]+	740.5594	740.5496	−9.8			
17	PC(33:3)	C_41_H_76_NO_8_P	[M+H]+	742.5387	742.5509	12.2			2.2
18	PC(33:2)	C_41_H_78_NO_8_P	[M+H]+	744.5543	744.5550	0.7			10.6
19	PC(O-34:1)	C_42_H_84_NO_7_P	[M+H]+	746.6064	746.5976	−8.8	184		15.2
20	PC(O-33:3)	C_41_H_78_NO_7_P	[M+Na]+	750.5414	750.5430	1.6		1.1	
21	PC(O-33:2)	C_41_H_80_NO_7_P	[M+H]+	752.5570	752.5501	−6.9			13
22	PC(34:4)	C_42_H_76_NO_8_P	[M+H]+	754.5387	754.5388	0.1			1.3
23	PC(32:0)	C_40_H_80_NO_8_P	[M+Na]+	756.5519	756.5451	−6.8			2.4
	PC(34:3)	C_42_H_78_NO_8_P	[M+H]+	756.5543	756.5451	−9.2			
24	PC(34:2)	C_42_H_80_NO_8_P	[M+H]+	758.57	758.5577	−12.3			2.4
26	PC(34:1)	C_42_H_82_NO_8_P	[M+H]+	760.5856	760.5847	−0.9	184		2.1
27	PC(O-36:5)	C_44_H_80_NO_7_P	[M+H]+	766.5751	766.5663	−8.8			11.3
28	PC(O-34:1)	C_42_H_84_NO_7_P	[M+Na]+	768.5883	768.5800	−8.3			14.5
	PC(O-36:4)	C_44_H_82_NO_7_P	[M+H]+	768.5907	768.5800	−10.7			
29	PC(35:3)	C_43_H_80_NO_8_P	[M+H]+	770.57	770.5699	−0.1			3.7
30	PS(O-34:0)	C_40_H_80_NO_9_P	[M+Na]+	772.5468	772.5480	1.2			5.4
31	PC(35:1)	C_43_H_84_NO_8_P	[M+H]+	774.6013	774.6147	13.4			56.5
32	PC(36:5)	C_44_H_78_NO_8_P	[M+H]+	780.5543	780.558	3.7			3.1
33	PC(36:4)	C_44_H_80_NO_8_P	[M+H]+	782.57	782.5715	1.5	184		1.3
34	PC(36:3)	C_44_H_82_NO_8_P	[M+H]+	784.5856	784.5753	−10.3			3.4
35	PC(36:2)	C_44_H_84_NO_8_P	[M+H]+	786.6013	786.6064	5.1			5
36	PC(36:1)	C_44_H_86_NO_8_P	[M+H]+	788.6169	788.6153	−1.6			8.9
37	PC(O-36:3)	C_44_H_84_NO_7_P	[M+Na]+	792.5883	792.5933	5.0			7.4
38	PC(37:5)	C_45_H_80_NO_8_P	[M+H]+	794.5700	794.5802	10.2			10.6
39	PC(37:4)	C_45_H_82_NO_8_P	[M+H]+	796.5856	796.5962	10.6	184		36.8
40	PC(36:3)	C_44_H_82_NO_8_P	[M+Na]+	806.5676	806.5585	−9.1			4.1
	PC(38:6)	C_46_H_80_NO_8_P	[M+H]+	806.5700	806.5585	−11.5			
41	PC(38:5)	C_46_H_82_NO_8_P	[M+H]+	808.5856	808.5864	0.8			5.8
42	PC(38:4)	C_46_H_84_NO_8_P	[M+H]+	810.6013	810.5938	−7.5	184		7.3

Up and down regulation are followed by C81/CEM and CEM/C81 abundance ratios, respectively.

*PC  =  diacyl glycerophosphocholine, PC O-  =  alkylacyl or alkenylacyl glycerophosphocholine.

As m/z 184 and 104 appeared in the LAESI spectra of the T lymphocytes and were also observed as collision induced dissociation (CID) products, the question of in-source fragmentation/degradation of lipids arises. When the LAESI spectrum was recorded for a standard lipid (PC(16∶0/18∶1)) under similar experimental conditions, the fragment ions m/z 104 and 184 were marginally observed (<0.5%). This confirms that the detected choline peaks correspond to metabolites and not to CID artifacts.

### Metabolite confirmation

**Figure 7:**
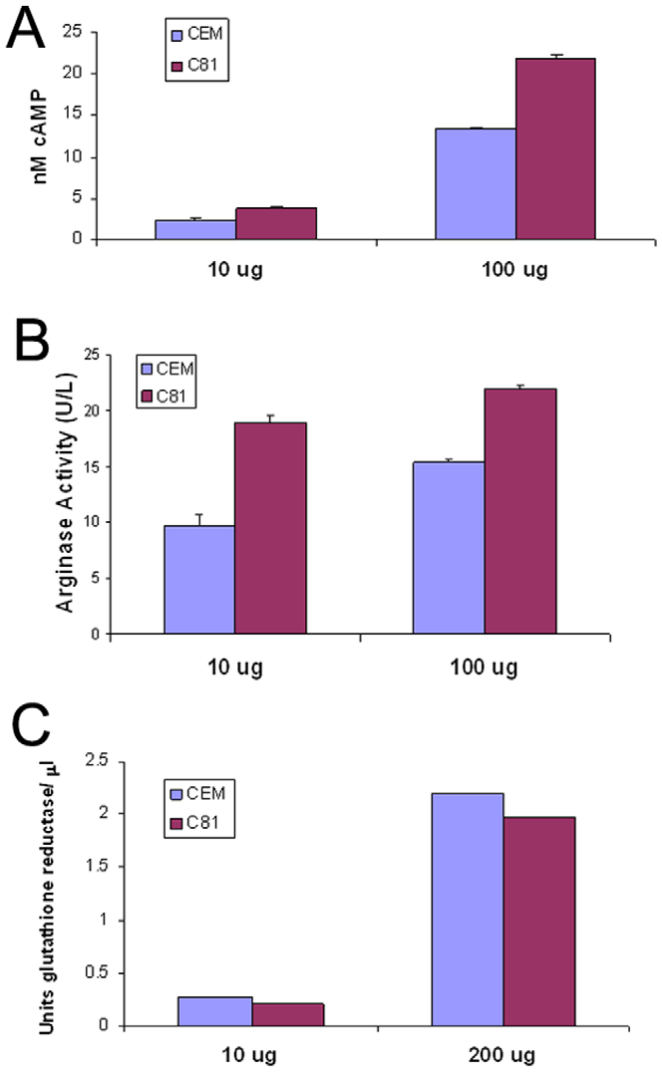
Confirmation of metabolic changes using conventional biochemical assays. A) Arginase activity was measured using the QuantiChrom Arginase Assay Kit (BioAssay Systems). CEM and C81 cell lysates (10 µg and 100 µg) were measured in triplicates. B) The concentration of cAMP was measured using the CatchPoint Cyclic-AMP Fluorescent Assay Kit (Molecular Devices). CEM and C81 cell lysates (10 µg and 100 µg) were measured in triplicates. C) Glutathione reductase from CEM and C81 cell lysates (10 µg and 200 µg) was measured utilizing the Glutathione Reductase Assay Kit (Sigma).

The observed changes in metabolite levels between non-HTLV1 transformed and HTLV1 transformed T lymphocytes were further verified at the protein level by quantifying the enzyme or protein involved in the related metabolic pathway. We measured the levels of cAMP, arginase, and glutathione reductase in CEM and C81 cells using biochemical assays. Arginase and cAMP levels were upregulated, whereas the glutathione reductase levels were downregulated in the HTLV1 transformed C81 cells compared to the non-HTLV1 transformed CEM cells (Figure 7). Therefore, these assay results correlated with the detected changes in the corresponding metabolites.

### H9 vs. H9-Tax1 and HUT102 cells

To determine if our observations were cell line specific and to explore the role of Tax1 expression, the LAESI experiments were extended to other cell lines, e.g., non-HTLV1 transformed T lymphocytes (H9), their Tax1-transfected counterparts (H9-Tax1) and HTLV1-transformed cells (HUT102 cells). The detected metabolites in these cells were found to be similar to the CEM and C81 cells with minor variations in the ion yields. The metabolic changes upon transfection are listed in Table 3. The pattern of down- and upregulation of key metabolites, such as glutathione and adenosine monophosphate, respectively, remained similar to the CEM/C81 case. These results suggest that the metabolic changes observed in the HTLV1 transformed cells can, in part, be attributed to Tax1 expression.

**Table 3:** Metabolite abundance ratios indicating up or down regulation for HTLV1 transformed T cells (C81, HUT102), and HTLV3 transfected and Tax1 or Tax3 expressing cells (293-HTLV3, H9-Tax1, 293-Tax3, respectively).

No.^a^	Metabolite (chemical formula)	CEM vs. C81		H9 vs. H9-Tax1		H9 vs. HUT102		293T vs. 293T-HTLV3		293T vs. 293T-Tax3	
		Up	Down	Up	Down	Up	Down	Up	Down	Up	Down
1	Thioacetamide (C_2_H_5_NS)	-	2.4	-	-	-	-	-	-	-	-
2	Putrescine (C_4_H_12_N_2_)	3	-	3.2	-	6.1	-	-	-	-	-
	Pyrrolidine/Degradation product **a**, (C_4_H_9_N)										
3	Choline (C_5_H_14_NO)	-	1.9	-	2.9	2.4	-	-	2.4	-	2.5
4	Proline (C_5_H_9_NO_2_)	-	2.1	-	10.1	1.2	-	-	-	-	-
5	Taurine (C_2_H_7_NO_3_S)	-	2.9	-	1.7	-	3.9	-	-	-	-
6	Creatine (C_4_H_9_N_3_O_2_)	4.6	-	2.3	-	-	-	-	2.0	1.6	-
7	Spermidine (C_7_H_19_N_3_)	1.7	-	1.0	-	1.3	-	2.0	-	2.2	-
8	p-Aminobenzoic acid (C_7_H_7_NO_2_)	2.7	-	-	1.7	-	1.2	-	3.6	3.4	-
9	Iminoaspartic acid (C_4_H_5_NO_4_)	-	1.6	-	6.0	1.4	-	-	2.4	1.9	-
10	Arginine (C_6_H_14_N_4_O_2_)	3.4	-	1.2	-	22.7	-	-	-	-	-
11	Dopamine (C_8_H_11_NO_2_)	3.0	-	-	1.4	2.5	-	-	-	-	-
12	Phosphocholine (C_5_H_14_NO_4_P)	-	4.8	-	4.1	1.2	-	-	4.0	-	1.5
13	Carbamoyl-phosphate CH_4_NO_5_P	-	2	-	3.5	3.4	-	-	-	-	-
14	Spermine (C_10_H_26_N_4_)	-	2.3	-	1.8	1.8	-	-	2.0	-	1.2
	Degradation product **c** (C_7_H_13_N)										
	Degradation product **b** (C_7_H_16_N_2_)										
15	Methoxytyramine (C_9_H_13_NO_2_)	-	5.3	-	3.7	-	4.7	-	5.0	2.9	-
16	N-acetyl aspartic acid/N-formyl glutamic acid (C_6_H_9_NO_5_)	2.1	-	1.7	-	3.9	-	4.8	-	2.9	-
17	Homovanillic acid (C_9_H_10_O_4_)	3.5	-	1.3	-	-	-	-	16.5	-	6.8
18	Glycerophosphocholine (C_8_H_20_NO_6_P)	-	2.4	-	6.1	25.6	-	-	3.5	-	4.4
19	Glutathione (C_10_H_17_N_3_O_6_S)	-	4.6	-	9.8	-	2.1	-	1.5	3.8	-
20	8-Hydroxyguanosine (C_10_H_13_N_5_O_6_)	-	1.9	-	-	-	-	-	-	-	-
21	Adenosine monophosphate (C_10_H_14_N_5_O_7_P)	7.6	-	3.1	-	21.5	-	-		-	-

a The metabolite numbers are according to the list provided in Table 1.

### 293T vs. 293T-HTLV3 and 293T-Tax3 cells

To determine the specificity of the observed metabolite changes to HTLV1 transformation, we performed LAESI experiments on non-HTLV3 transformed 293T kidney epithelial cells, and on HTLV3 and Tax3 transfected 293T cells. Although the 293T cells showed a variety of ions that were not detected in the CEM, C81, H9 and HUT102 cells (see Table 4), there were some metabolites common to all these cells (see Table 3). The observed changes for HTLV3 and Tax3 transfected 293T cells did not match those found in HTLV1 transformed cells. For example, the lipid peaks that showed prominent changes in the HTLV1 transformed cells were found to be unaltered in HTLV3/Tax3 affected 293T cells (data not shown).

**Table 4:** Additional metabolites detected in 293T, 293T-HTLV3 and 293T-Tax3 cells, and their abundance ratios indicating up and down regulation due to HTLV3 transfection or the presence of Tax3.

Additional metabolites detected in 293T, 293T-HTLV3 and 293T-Tax3 cells					293T vs. 293T-HTLV3		293T vs. 293T-Tax3	
No.	Metabolite	Ion	Measured mass	Error (mDa)	Up	Down	Up	Down
22	5-Aminoimdazole (C_3_H_5_N_3_)	[M+H]^+^	84.0705	14.3	-	2.2	1.7	-
23	Dimethyl sulfide (C_2_H_6_S)	[M+Na]^+^	85.0097	0.9	1.9	-	2.2	-
24	Sarcosine/Alanine (C_3_H_7_NO_2_)	[M+H]^+^	90.0525	-3	-	2.5	-	1.1
25	Glycerol (C_0_H_8_O_3_)	[M+Na]+	115.021	−16.1	8.0	-	-	1.6
26	Glutarate semialdehyde (C_5_H_8_O_3_)	[M+H]^+^	117.0277	−27.5	2.5	-	3.1	-
27	Succinic acid (C_4_H_6_O_4_)	[M+H]^+^	119.0276	−6.8	-	3.3	-	1.5
28	Amino malonic acid (C_3_H_5_NO_4_)	[M+H]+	120.0118	−17.3	2.7	-	4.8	-
29	Homoserine/threonine (C_4_H_9_NO_3_)	[M+H]^+^	120.0803	14.2	-	3.0	-	2.9
30	Creatinine (C_4_H_7_N_3_O)	[M+Na]^+^	136.0486	−0.1	3.7	-	2.7	-
31	Betaine (C_5_H_11_NO_2_)	[M+K]^+^	156.0464	3.7	-	3.2	2.2	-
32	2-Aminomuconic acid semialdehyde (C_6_H_7_NO_3_)	[M+Na]^+^	164.0272	−5.2	-	1.9	3.0	-
33	Mannitol/Sorbitol (C_6_H_14_O_6_)	[M+Na]^+^	205.0655	−3.3	-	36	-	20

## Discussion

In this study we detected the changes in various metabolite and lipid levels upon HTLV transformation and Tax expression in T lymphocytes and kidney epithelial cells. Among the metabolites identified, glutathione, spermine, choline, phosphocholine, glycerophosphocholine, thioacetamide, proline, taurine, carbamoyl phosphate, methoxytyramine and 8-hydroxy guanosine were downregulated in the C81 vs. CEM cells, whereas the levels of creatine, arginine, dopamine, homovanillic acid and AMP were upregulated. Some of the key metabolic changes detected in C81 cells were also observed in H9-Tax1 vs. H9 and HUT102 vs. H9 cells. These metabolites participate in several biochemical pathways, such as polyamine biosynthesis, creatine biosynthesis, AMP biosynthesis, dopamine metabolism, lipid metabolism, redox reactions etc. Their biological importance and relevance to transformation and/or Tax expression is discussed below.

### HTLV1 transformation specific metabolites

There were a few metabolites, including putrescine, taurine, arginine, and adenosine monophosphate, that were differentially regulated in HTLV1 transformed cells. These metabolites were not detected in HTLV3 or Tax3 transfected cells, indicating that they were specific to HTLV1 induced transformation.

**Figure 8:**
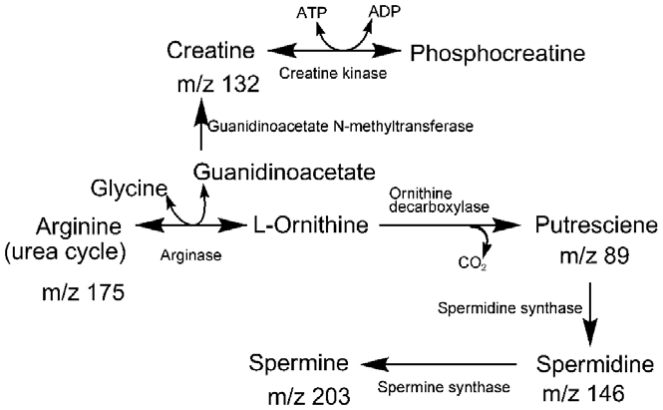
Metabolites in the creatine and polyamine biosynthesis pathways detected by LAESI-MS in T lymphocytes.

Putrescine, along with spermine and spermidine belong to the polycationic compounds named polyamines [46] present in all living cells. Due to electrostatic interactions between the positively charged ammonium groups of the polyamines and the negatively charged phosphates of nucleic acids, they often associate. They are involved in genetic processes such as DNA synthesis and gene expression and play a major role in cell proliferation, cell differentiation, and programmed cell death. The biosynthesis of polyamines is tightly regulated in cells, and ornithine in the urea cycle is their precursor (Figure 8). Therefore, the levels of each polyamine are linked to the actual status of the cell. In the present study, spermine levels were found to be somewhat lower in all the transformed cells (except for the case of HUT102), whereas the levels of putrescine and spermidine, the precursors of spermine, were upregulated in HTLV1 transformed cells. Spermidine was also upregulated in the 293T-HTLV3 and the 293T-Tax3 cell lines. This reveals that the expression of full length HTLV3 or just Tax3 affects the tightly regulated biosynthesis of polyamines in the cells, thereby causing disturbances in the genetic processes. Although the trends for individual amines were not completely consistent among HTLV1 transformed cells, the effect of viruses on the overall polyamine biosynthesis is clearly reflected. Further studies are required to explore whether the individual amine levels correlate with the stage of the viral transformation.

Interestingly, arginine that is converted into ornithine, the precursor of polyamines in the urea cycle, was also upregulated in HTLV1 transformed cells (quite dramatically for HUT102). Upregulation of arginase in transformed cells as detected by the enzyme assay (Figure 7B), is consistent with the elevated levels of arginine upon viral transformation. Deprivation of arginine causes serious disturbances in cellular function and enhances apoptosis [47]. The role of arginine in the survival of endothelial cells during oxidative stress has also been demonstrated [48]. A recent report shows regulation of T lymphocyte function in cancer by arginine availability [49]. Arginine is also a precursor in the biosynthesis of creatine (Figure 8), an important molecule in energy supply. Prevalence of abnormal creatine phosphokinase levels in the blood of HTLV2 infected patients has been reported [50]. Recently, it was shown that the enzymes related to creatine and arginine metabolism were found to be significantly upregulated in malignant cells [51]. Finding upregulation of arginine and putrescine in HTLV1 transformed cells highlights the importance of polyamine and creatine biosynthesis for energy production to support the high rates of cellular proliferation that is common for transformed cells.

Levels of phosphorylated adenosine nucleotides, including ATP, ADP and AMP, define the energy state in living cells. Quantitation of individual adenine nucleotides is frequently used for the assessment of the energy state of cells [52], [53], [54]. The level of exogenous ATP in the body may be increased in various inflammatory and shock conditions. The importance of extracellular ATP for cell-to-cell communication and in the immune system is known [55], [56], [57]. Classical HPLC based metabolomic technologies have been used to detect and quantify these molecules [13], [54], [58].

**Figure 9:**
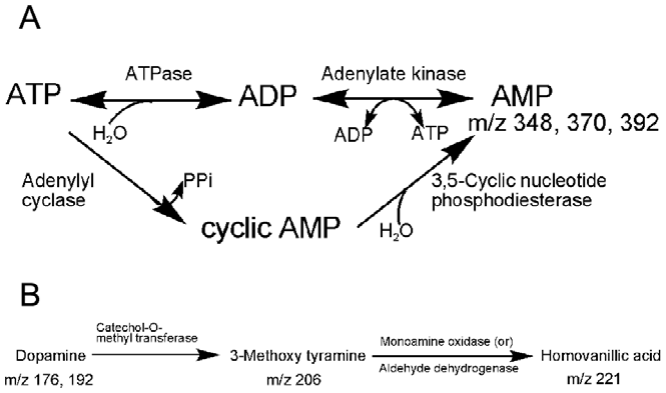
Metabolites related to A) adenosine monophosphate (AMP) and B) dopamine detected by LAESI-MS in T lymphocytes.

Here we detected AMP directly from cells using the LAESI technique. Significantly elevated AMP abundance was observed in HTLV1 and Tax1 transformed cells. AMP can be formed by the dephosphorylation of ATP/ADP or by the hydrolysis of cAMP (Figure 9A). Apart from being a degradation product of ATP, AMP is known to activate the AMP-activated kinase (AMPK) system that is ubiquitously expressed in mammalian cells. It is involved in the response to a variety of metabolic stresses that disturb the cellular energy homeostasis [59], [60], [61]. The cause of elevated AMP levels in HTLV1 and Tax1transformed cells requires further investigation.

cAMP is a second messenger and activates several protein kinases that are involved in significant biochemical processes. The amount of cAMP known to be present in T lymphocytes is ∼6 pmol/10^7^ cells. We had difficulty in the detection of cAMP by LAESI-MS due to an overwhelming spectral interference from glutathione. A mass spectrometer with better resolving power could separately detect these two species. We applied an alternative immunoassay method to measure the levels of cAMP (Figure 7). The cAMP levels (adenylyl cyclase activity, Figure 9A) were increased in HTLV1 transformed cells compared with non-HTLV1 transformed cells. Importantly, targeting of cAMP pathway has been explored as a potential therapeutic option for the treatment of leukemia/lymphoma and therefore, could also be useful for the treatment of ATL [62]

We identified dopamine, a neuromodulator, and its metabolites, methoxytyramine and homovanillic acid, in the studied T lymphocytes. Dopamine belongs to the group of catecholamines, and is involved in the neuroimmunological network [63]. T lymphocytes can be activated by neurotransmitters via neurotransmitter receptors that can elicit crucial functions [64]. Synthesis of catecholamines in mouse lymphocytes, and their increased levels in the activated state was noticed earlier [63], [65]. Dopamine can be biosynthesized in the body from tyrosine, and the related metabolic pathways are also well established (see, e. g., Figure 9B). In the present study we found upregulation of dopamine and homovanillic acid levels and downregulation of methoxytyramine in HTLV1 transformed cells. To our knowledge changes in these metabolites have never been documented for HTLV transformed cells. Considering their implications in neuronal processes, it would be interesting to determine if they were also deregulated in HAM/TSP patient samples and understanding how changes in their expression relate to this diseased state.

### 293T vs. 293T-HTLV3 metabolites

Transfection of 293T cells with HTLV3 molecular clone resulted in the downregulation of many of the metabolites detected in the HTLV1 transformed cells. In fact, only two metabolites (spermidine and N-acetyl aspartic acid) were upregulated in both HTLV1 transformed and HTLV3 transfected cells. There were additional metabolites detected (see Table 4), which are likely to be reflective of cell type differences, as they were only found in the 293T cell experiments.

One particularly interesting metabolite is glutathione. Glutathione was downregulated in HTLV3 transfected cells as well as HTLV1 transformed cells, indicating that downregulation of glutathione is a consequence of viral infection, not necessarily the transformation process. The reduced form of glutathione (GSH) is the most predominant thiol present in mammalian cells with concentrations up to 12 mM [66]. GSH serves several important functions, such as antioxidant (protection against oxidative stress), cofactor in isomerization reactions, transport and storage form of cysteine, and regulator of intracellular redox status, cell proliferation and apoptosis [67], [68], [69]. Biologically the oxidized glutathione (GSSG) is converted to GSH by the enzyme glutathione reductase. The ratio of GSH and GSSG serves as a representative marker of the antioxidative capacity of the cell [70]. Cellular GSH concentrations are markedly reduced in response to protein malnutrition, oxidative stress, and many pathological conditions and can sensitize cells to apoptosis. In fact, reducing GSH levels through buthionine sulfoximine treatment, resulted in increased sensitivity of HTLV1 transformed cells to 13-cis-retinoic acid induced cell death [71].

### Metabolite changes due to Tax3 expression

When comparing metabolite levels between HTLV3 and Tax3 transfected 293T cells, there were a number of common changes observed, including decreased choline, phosphocholine, spermine, homovanillic acid, and glycerophosphocholine and increased spermidine and N-acetyl aspartic acid. These results indicate that the lipid metabolism pathway as well as the creatine and polyamine biosynthesis pathways are commonly deregulated after expression of HTLV3 and Tax3, indicating that the noted changes are likely due to Tax3 expression.

N-acetyl aspartic acid or N-acetylaspartate (NAA) is a novel metabolite that is upregulated in all cell types and all conditions tested. NAA levels are well known to be altered in diseases such as Alzheimer's, epilepsy and schizophrenia [72]. In addition, the abundance of NAA in the urine is a marker for Canavan disease (CD), which is a leukodystrophy caused by deficiency of the enzyme aspartoacylase [73]. The expression of NAA is associated with neuronal processes and thus the detection in lymphocytes is unexpected. Interestingly, HTLV1 infection can result in the neurological disorder HAM/TSP. HAM/TSP is a demyelinating disorder and can results in the degradation of the cervical spinal cord and the brainstem [74]. Therefore, the upregulation of NAA may be reflective of viral induced changes that could have important implications for HAM/TSP.

### Choline containing metabolites including lipids

**Figure 10:**
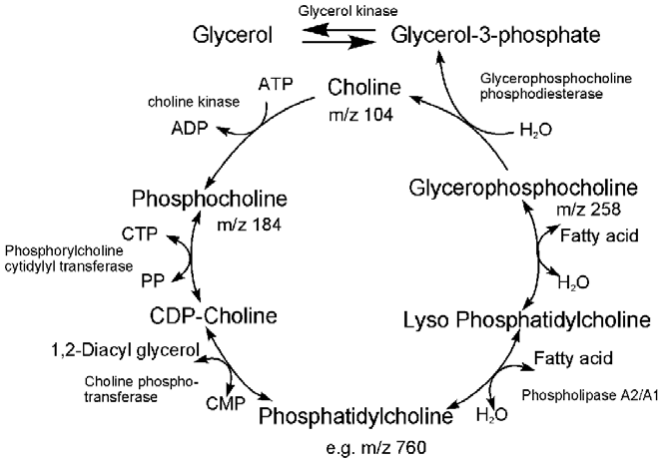
Metabolites in the lipid metabolism pathway detected by LAESI-MS in T lymphocytes.

We found choline containing metabolites, i.e., choline, phosphocholine, glycerophosphocholine, and several glycerophosphocholine lipids in the LAESI spectra of T lymphocytes and kidney epithelial cells. Except for the HUT102 case, when compared to non-transformed cells, all these metabolites were downregulated upon transformation by HTLV1, HTLV3, Tax1 or Tax3. The role of these metabolites in lipid metabolism is shown in Figure 10.

Usually, choline containing metabolites are monitored by *in vivo* NMR spectroscopy, but with this technique it is difficult to determine which specific metabolites are altered [14]. Using LAESI MS enabled the direct monitoring of choline containing metabolites. The LAESI spectra of non-transformed and transformed cells provided information on both the precursors and lipid components simultaneously. We also found a decrease in the glycerophosphocholine lipid content in HTLV1 transformed cells confirming increased lipid catabolism to produce fatty acids. Apart from a few lipids (PC(30∶0), PC(32∶5), PC(32∶3), PC(32∶0), and PC(34∶6) that remained at higher levels in HTLV1 transformed cells, most of the glycerophosphocholine lipids present in non-HTLV1 transformed cells were downregualated in the HTLV1 transformed ones.

### Conclusions and future directions

We used the LAESI technique to identify metabolic changes in HTLV1 and Tax1 transformed T lymphocytes and in HTLV3 and Tax3 transfected kidney epithelial cells. We found virus type specific (HTLV1 vs. HTLV3), expression specific (Tax1 vs. Tax3) and cell type specific (T lymphocytes vs. kidney epithelial cells) changes in the metabolite profiles. We have identified a number of metabolites that are known in the literature to be deregulated in the viral transformation process (e. g., arginine, cAMP, glutathione) as well as multiple novel metabolites that may have implications in HTLV1-induced transformation (e. g., putrescine, N-acetyl aspartic acid, methoxytyramine). These new findings point to metabolic pathways that have a heretofore unexplored role in the viral transformation of host cells. Future studies will focus on the elucidation of the mechanism of metabolite deregulation and its consequences in terms of disease progression and treatment options.

Our results also demonstrate the application of a new technique, LAESI-MS, which is capable of in situ detection of metabolites and lipid components from the entire volume of the cells. Because LAESI uses the native water content of the cells or tissue to couple the laser energy into the sample, it does not require extensive sample preparation or the application of a matrix material.

Although cell populations are used for this study, the technique can also be applied for analyzing intracellular metabolites from a single cell. The LAESI method has already been utilized for the analysis of metabolites from large single cells (e. g., sea urchin eggs of 90 µm diameter) [43]. Since the T lymphocytes are significantly smaller in size (10–15 µm), modifications in the sample handling, for example, the use of micromanipulators, and reduction in the laser spot size are required. Such advances are required to explore cell-to-cell metabolic variations, as well as cells at different stages of the cell cycle.
